# Prognostic Nutritional Index Predicts Outcome of PD-L1 Negative and MSS Advanced Cancer Treated with PD-1 Inhibitors

**DOI:** 10.1155/2022/6743126

**Published:** 2022-06-06

**Authors:** Yan Zhang, Jun Jin, Min Tang, Ping Li, Li-Na Zhou, Yi-Ping Du, Min-Bin Chen

**Affiliations:** Department of Oncology & Radiotherapy, Affiliated Kunshan Hospital of Jiangsu University, Jiangsu, China

## Abstract

**Purpose:**

Tumor mutational burden (TMB), microsatellite instability-high (MSI-H), and expression of programmed death ligand-1 (PD-L1) have emerged as predictive biomarkers for responsiveness to immune checkpoint inhibitors (ICIs) in several cancer types. However, for patients with negative PD-L1 expression, or microsatellite stability (MSS), some cases may experience favorable response to immunotherapy, and there is currently a lack of good relevant predictors. We tried to introduce several peripheral blood markers for predicting treatment outcome and immune-related adverse events (irAEs) in PD-L1 negative and MSS patients.

**Methods:**

A retrospective study of 142 PD-L1 negative and MSS patients was carried out. The association of peripheral blood markers including lactate dehydrogenase (LDH), neutrophil-to-lymphocyte ratio (NLR), platelet-to-lymphocyte ratio (PLR), albumin-to-globulin ratio (AGR), prognostic nutrition index (PNI), and other factors with clinicopathological characters and prognosis were assessed by Cox regression and Kaplan-Meier methods.

**Results:**

Lower level of PNI and poor performance status (ECOG score of 2) was correlated with significantly shorter overall survival (OS) and worse outcome of ICIs. The multivariate analysis revealed that PNI (for OS HR = 0.465, 95% CI: 0.236–0.916, *p* = 0.027; for PFS HR = 0.493, 95% CI: 0.251–0.936, *p* = 0.031) and ECOG score (for OS HR = 4.601, 95% CI: 2.676–7.910, *p* < 0.001; for PFS HR = 2.830, 95% CI: 1.707–4.691, *p* < 0.001) were independent prognostic factors for OS and PFS. NLR was related to the onset of irAEs.

**Conclusions:**

Pretreatment level of PNI and NLR, beyond PD-L1 expression and MSS, can improve the predictive accuracy for immunotherapy outcomes and has the potential to expand the candidate pool of patients for treatment with ICIs.

## 1. Introduction

The use of immune checkpoint inhibitors (ICIs) targeting programmed cell death receptor (PD-1) or its ligand (PD-L1) alone or in combination with chemotherapy is a promising cancer treatment strategy [1, 2]. However, as real-world experience confirmed only a minority of patients respond to immunotherapy and have improved long-term survival [3, 4]. It is critical to identify reliable predictive biomarkers for selecting patients who can benefit from ICIs.

PD-L1 status is recognized as the most validated factor predicting immunotherapy outcome. PD-L1 is a cell surface protein expressed physiologically in a variety of tissues. Elevated PD-L1 expression on tumor cells or tumor-infiltrating lymphocyte (TIL) results in the exhaustion of T cells, thus, the attenuated tumor-specific immunity promoting tumor progression [5, 6]. Regarding that some PD-L1 negative patients respond to PD-1/PD-L1 inhibitors and some PD-L1 positive patients do not, it was far from a perfect biomarker [7, 8].

Microsatellite instability (MSI) and tumor mutational burden (TMB) were another two potential predictive factors for ICIs. MSI-H causes a buildup of somatic mutations in tumor cells and leads to a spectrum of molecular and biological changes including high tumor mutational burden, increased expression of neoantigens, and abundant tumor-infiltrating lymphocytes [9, 10]. MSI and TMB are independent of PD-L1 status in most cancer types, although the complementary utilization of TMB, PD-L1, and MSI-H has the potential to predict ICIs responsiveness better than each alone. For patients with negative PD-L1 expression and/or MSS, some patients may still receive immunotherapy and achieve a certain effect. For these patients, there is currently a lack of good relevant predictive indicators.

Host inflammation or immune-nutritional index have attracted attention as prognostic factors to predict response to anticancer drugs [11]. The prognostic value of some inflammation-related peripheral blood parameters which may reflect the balance between nonspecific inflammation and immunoreaction have been taken into account. The previous studies by our team have demonstrated neutrophil-to-lymphocyte ratio (NLR) and platelet-to-lymphocyte ratio (PLR) as well as the albumin-to-globulin ratio (AGR) and the prognostic nutrition index (PNI) were potential markers for predicting prognosis in patients with gastric cancer [12, 13]. Compared to other factors, peripheral blood markers are more economical and practical. However, the predictive value of these biomarkers for immunotherapy remains to be fully elucidated.

We conducted this retrospective study to explore the associations between immunonutrition-related peripheral blood markers including NLR, PLR, lymphocyte (LYM), AGR, PNI, lactate dehydrogenase (LDH), and prognosis of PD-L1 negative and MSS cancer patients treated with immunotherapy.

## 2. Materials and Methods

### 2.1. Study Population

The prospective observational study included 149 patients with a histologically or cytologically proven diagnosis of malignant neoplasms. Immunohistochemistry (IHC) results confirmed PD-L1 negative expression and next-generation sequencing identified MSS patients. All the cases were treated with anti-PD-1/PD-L1 antibodies with or without chemotherapy until disease progression, discontinuation by treatment-related adverse events, or death at Affiliated Kunshan Hospital of Jiangsu University from October 2018 to May 2021. Patients with any of the following were excluded from this study: the second primary tumor, active concurrent infection, autoimmune disease, and incomplete follow-up data. The study was performed in agreement with the guidelines of the Declaration of Helsinki. It was reviewed by Institutional Review Board of Affiliated Kunshan Hospital of Jiangsu University, and every patient enrolled has provided written informed consent.

### 2.2. Data Collection

The clinical characteristics of the patients including age, sex, pathologic type, performance status, treatment, and hematologic examination were available on the clinical records. The NLR was defined as the absolute neutrophil count divided by the absolute lymphocyte count. In the same way, PLR was calculated by dividing the absolute platelet count by the absolute lymphocyte count. The AGR and PNI were calculated using the following equations as previously reported:AGR = albumin/(total protein − albumin) and PNI = albumin (g/L) + 5∗total lymphocyte count (10^9^/L). Overall survival (OS) was defined as the interval between initial immunotherapy to the date of death or last follow-up. Progression-free survival (PFS) was from the first day of treatment with ICIs to the time of progression, relapse, death, or last follow-up.

### 2.3. Statistical Analysis

The optimal cutoff values of LYM, LDH, NLR, PLR, AGR, and PNI were estimated by the receiver operating characteristics (ROC) curve (data not shown). The Kaplan–Meier method and log-rank tests were used to describe the OS and PFS curve and compare survival rate. Univariate and multivariate analyses were also performed to assess the hazard ratios (HRs) for independent prognostic values of the covariates. All the statistical analyses were performed using SPSS version 16.0 (SPSS, Chicago, IL). A 2-sided *p* value of less than 0.05 was considered as significant.

## 3. Results

### 3.1. Patient Characteristics

Totally, 142 cancer patients of stage IIIB-IV were enrolled in our study.  Table 1 demonstrated the characteristics of the patients. The median age was 64.0 years old (range from 23 to 85 years), and 75 (52.8%) patients were male. The most common cancer type was lung cancer (28.17%), followed by colorectal cancer (14.79%) and gastric cancer (12.68%). 76.76% patients showed good performance status (ECOG score of 0-1). High TMB was defined as more than 20 mut/Mb (42.96%), TMB-intermediate as 6-19 mut/Mb (42.25%), and TMB-low as fewer than 5 mutations/Mb (14.79%). The cutoff value of LDH, LYM, NLR, PLR, AGR, and PNI was 240, 1.28, 3.18, 201, 1.24, and 50.03, respectively, when the Youden index was maximal.

### 3.2. Prognostic Factors for OS and PFS

**Figure 1:**
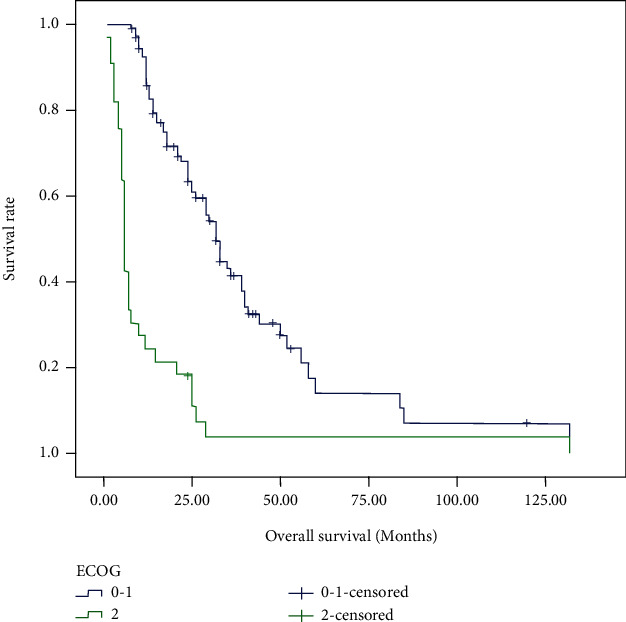
Kaplan-Meier survival curves of overall survival according to ECOG score.

**Figure 2:**
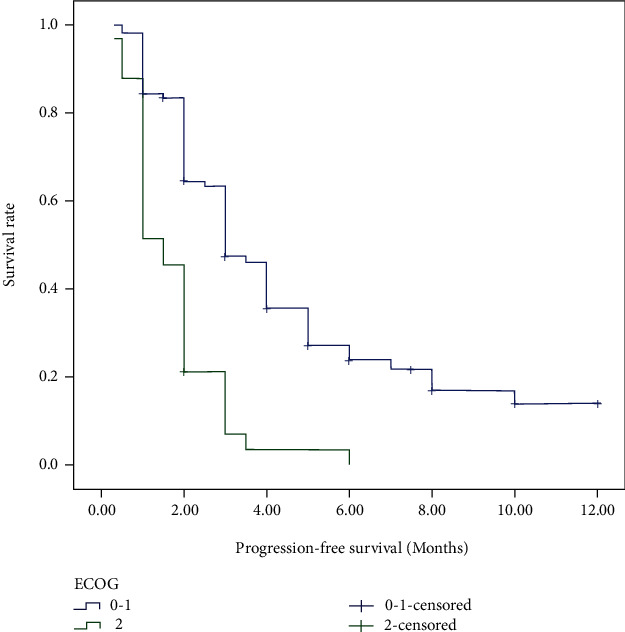
Kaplan-Meier survival curves of progression-free survival according to ECOG score.

**Figure 3:**
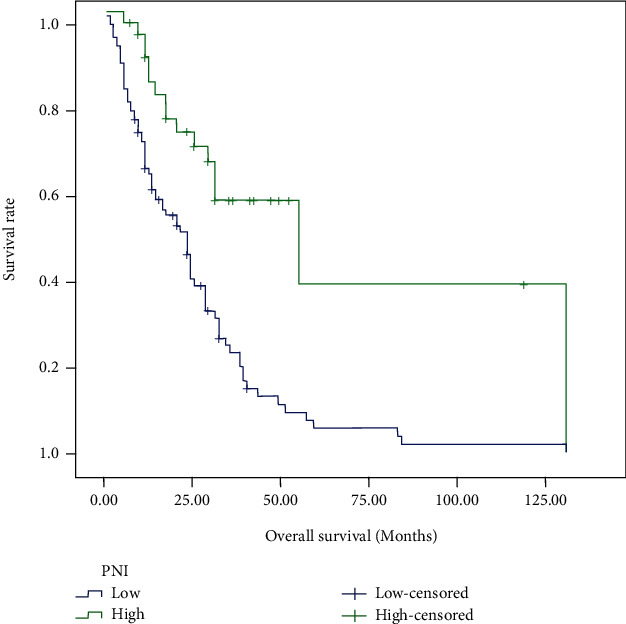
Kaplan-Meier survival curves of overall survival according to prognostic nutrition index.

**Figure 4:**
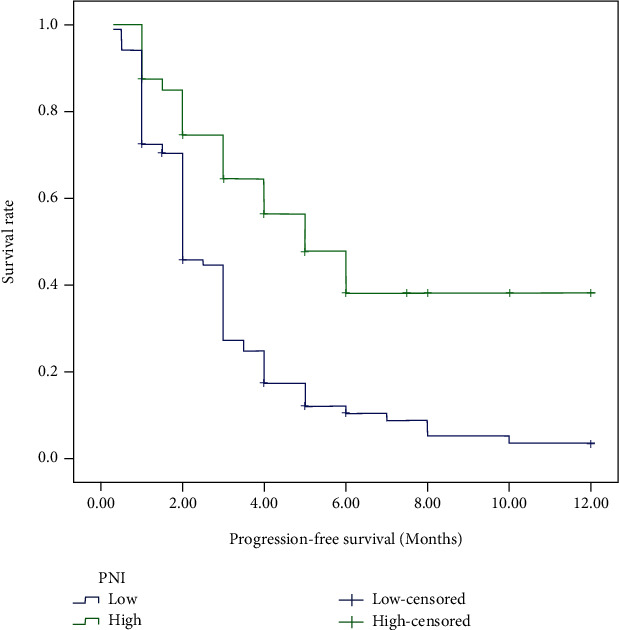
Kaplan-Meier survival curves of progression-free survival according to prognostic nutrition index.

For the cases in this study, 48 (33.80%) patients were still alive at the last follow-up. The median OS for all patients was 19.0 months, and the median PFS of ICIs was 2.0 months. Tables 2 and 3 demonstrated the prognostic effect of the clinical factors. According to the univariate analysis, ECOG score of 2 (*p* < 0.001) and higher NLR (*p* = 0.046) were identified as poor prognostic factors for OS. Higher lymphocyte count (*p* = 0.006) and higher PNI (*p* < 0.001) predicted longer OS. Female (*p* = 0.003), as well as patients with good performance status (*p* < 0.001), higher lymphocyte count (*p* = 0.011), higher AGR (*p* = 0.004), and PNI (*p* < 0.001) had longer PFS after immunotherapy. Cox regression model was used to conduct multivariate analysis. Performance status and PNI were verified as independent prognostic factors for OS and PFS. As shown in Figures 1–4, median OS and PFS were shorter in poor performance status group (OS: 6.0 months vs. 24.0 months, *p* < 0.001; PFS: 1.25 months vs. 2.0 months, *p* < 0.001) and low-PNI group (OS: 14.5 months vs. 28.0 months, *p* < 0.001; PFS: 2.0 months vs. 3.0 months, *p* < 0.001).

The most frequently enrolled cancer type in this study was lung cancer. We further analyzed the information of 40 lung cancer patients. There were 17 (42.5%) female and 23 (57.5%) male patients were analyzed, and the median age was 64.0 years. Classified by the pathologic types, 5 (12.5%) were small cell lung carcinoma (SCLC), 28 (70%) were pulmonary adenocarcinoma, and 7 (17.5%) were squamous cell carcinoma (SCC). According to the results of multivariate analysis, ECOG score rather than PNI was an independent prognostic factors for OS and PFS (for OS HR = 7.003, 95% CI: 1.409–34.813, *p* = 0.017; for PFS HR = 5.402, 95% CI: 1.427–20.455, *p* = 0.013) in lung cancer patients.

### 3.3. Factors Associated with Immune-Related Adverse Events (irAEs)

**Figure 5:**
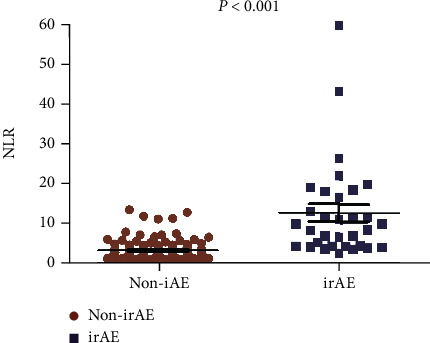
Comparison of neutrophil-to-lymphocyte ratio levels according to the onset of irAEs.

Among all the cases, 22.54% patients suffered from irAEs. Most of the irAEs were 1-2 grade, including rash (10.56%), reactive cutaneous capillary endothelial proliferation (RCCEP) (7.04%), hypothyroidis (5.63%), and enteritis (2.11%). The most common severe irAE was checkpoint inhibitor pneumonitis (CIP) with the incidence of 3.52%. It is found that NLR level may be associated with the incidence of irAEs. As indicated in Figure 5, patients suffering from irAEs had a higher level of NLR (*p* < 0.001).

## 4. Discussion

Although cancer treatment is experiencing a revolution with the emergence of ICIs, biomarkers for predicting prognosis of immunotherapy are obscure [14, 15]. Candidate biomarkers like PD-L1 and MSI had types of limitations in clinical use. This study mainly evaluated the prognostic value of peripheral blood markers in advanced cancer patients treated with ICIs.

We observed that cancer patients with higher ECOG score and lower level of PNI predicted poor OS and worse immunotherapy outcomes. Moreover, patients with higher level of pretreatment NLR were more likely to suffer from irAEs. The findings may tell two important points. First, nutritional status and performance status are important prognostic markers for cancer patients. Second, the incidence of irAEs may be associated with excessive inflammatory response. These findings were in parallel with previous reports. Peng et al. [16] detailly descripted that in patients with advanced NSCLC treated with PD-1 inhibitors, pretreatment NLR, LDH, and PNI were independent predictive markers of OS and PFS. PNI and NLR were associated with the onset of irAEs.

The Eastern Cooperative Oncology Group (ECOG) performance status has been established as one of the most powerful independent prognostic factors in advanced NSCLC since it is a strong predictor of survival and adverse events [17]. Many clinical trials have excluded patients of ECOG score more than 2. Though PD-1/PD-L1 inhibitors appear to be well-tolerated, our study still showed that poor performance status patients had worse outcomes. PNI is a biomarker based on serum albumin level and total lymphocyte count. Several evidences reported that PNI reflected the systemic immunonutritional status and was a prognostic indicator in various cancers, including gastric cancer [13, 18], nasopharyngeal carcinoma [19], and lung cancer [20, 21]. It was easily calculated in daily routine. In our study, we found patients with higher level of pretreatment PNI or good performance status had longer OS and PFS. These patients showed good reserve function and were able to endure the immunotherapy. In other words, a decreased PNI means both malnutrition status and weak lymphocyte-mediated antitumor immune response, which may both contribute to disease progression and poor prognosis [22, 23]. Malnutrition and poor performance status result in a mass of negative consequences, such as impaired immune functions and quality of life (QOL), a higher degree of treatment-related toxicity, delayed cancer treatment, lower activity level, and shortened survival [21, 24].

While ICIs represent a new method against cancer, they have also produced a unique set of irAEs that could have serious or even fatal consequences. irAEs are independent toxicity caused by the nonspecific activation of the immune system and can affect almost all tissues and organs [25]. It increased the risk of hospitalization and the costs of treatment. However, some studies confirmed that patients who experienced irAEs had better PFS compared with those who had no irAEs [26, 27]. irAEs and tumor suppression may share common mechanisms of the activated immune system [28]. More and more studies were conducted to identify biomarkers associated with occurrence of irAEs. de Malet et al. [29] reported CD8-positive lymphocytes infiltrated in tissues with irAEs and activation of lymphocytes play a central role in the development of irAEs. The results of our study were similar with the conclusions of Matsukane et al. [30] that the elevation of the NLR was correlated with the onset and severity of irAEs.

In our study, the most common used biomarkers of TMB for predicting immunotherapy outcome did not show stable predictive value. We suspect that immune system and tumor microenvironment (TME) were extremely complex, involving multiple cells and substances. ICIs regulated not only cytotoxic T cells but also regulatory T cells, macrophages, helper T cells, natural killer cells, dendritic cells, and bone marrow-derived suppressor cells [31]. A single factor could not perfectly and systematically reflect the immunity, inflammation, and nutritional status of the person. We need to identify more factors and consider their combined effects.

There were several limitations to the present study. It could not deny that it was a retrospective study, and our sample size was relatively small. All the patients enrolled were coming from a single institution. We also excluded the patients with incomplete follow-up data, which may lead to selection bias because several patients with no response were easily in this group. Prospective studies or multicenter studies are needed to validate our results. Moreover, though we found that peripheral blood markers like PNI and NLR could predict clinical outcome and irAEs, we assessed only the pretreatment level of these factors. It can be understood that nutritional status and inflammation may change due to treatment, but the dynamic changes were not recorded. The most important of all, a prognostic scoring system should be established that multiple information including nutrition, inflammation, and immunity would be provided to enrich the predictive system.

## 5. Conclusion

In conclusion, our data demonstrated the ECOG score, pretreatment level of peripheral blood markers PNI was correlated with survival and treatment outcome; NLR was associated with the onset of irAEs in cancer patients receiving ICIs. These indicators were easily calculated and useful in clinical practice. If our results are further validated, peripheral blood markers may be used as tools to identify patients that can benefit from ICIs. We may attach importance to performance status and nutrition status of cancer patients to improve outcomes following immunotherapy and prolong their survival.

## Figures and Tables

**Table 1:** Characteristics of the enrolled patients.

Characteristics	*N* (%)
Age (years)	
<60	56 (39.44)
≥60	86 (60.56)
Sex	
Male	75 (52.80)
Female	67 (47.20)
ECOG (score)	
0-1	109 (76.76)
2	33 (23.24)
Tumor location	
Lung cancer	40 (28.17)
Colorectal cancer	21 (14.79)
Gastric cancer	18 (12.68)
Breast cancer	6 (4.23)
Esophageal cancer	9 (6.34)
Pancreatic cancer	8 (5.63)
Gynecologic cancers	14 (9.86)
Urologic cancers	6 (4.23)
Others	20 (14.08)
LDH (U/L)	
<240	72 (50.70)
≥240	70 (49.30)
LYM (∗10^9^/L)	
<1.28	73 (51.41)
≥1.28	69 (48.59)
NLR	
<3.18	68 (47.89)
≥3.18	74 (52.11)
PLR	
<201	80 (56.34)
≥201	62 (43.66)
AGR	
<1.24	54 (38.03)
≥1.24	88 (61.97)
PNI	
<50.03	102 (71.83)
≥50.03	40 (28.17)
TMB	
Low	61 (42.96)
Intermediate	60 (42.25)
High	21 (14.79)
irAEs	
No	110 (77.46)
Yes	32 (22.54)

**Table 2:** Univariate and multivariate analyses of biomarkers for overall survival.

Characteristics	Univariate analysis	*p* value	Multivariate analysis	*p* value
	HR (95% CI)		HR (95% CI)	
Age (years)				
<60	1.000		1.000	
≥60	1.880 (0.581-3.343)	0.546	0.913 (0.564-1.478)	0.711
Sex				
Male	1.000		1.000	
Female	0.868 (0.579-1.301)	0.493	0.468 (0.209-1.094)	0.177
ECOG				
0-1	1.000		1.000	
2	4.382 (2.809-6.837)	**<0.001**	4.601 (2.676-7.910)	**<0.001**
LDH				
<240	1.000		1.000	
≥240	1.172 (0.785-1.750)	0.438	1.721 (1.453-2.147)	0.167
LYM				
<1.28	1.000		1.000	
≥1.28	0.562 (0.371-0.851)	**0.006**	0.955 (0.528-1.727)	0.878
TMB				
Low	1.000		1.000	
Intermediate	0.958 (0.655-1.401)	0.824	1.103 (0.741-1.722)	0.571
High	0.812 (0.610-1.018)	0.153	0.754 (0.540-1.054)	0.098
NLR				
<3.18	1.000		1.000	
≥3.18	1.533 (1.007-2.335)	**0.046**	1.177 (0.625-2.216)	0.615
PLR				
<201	1.000		1.000	
≥201	1.444 (0.954-2.186)	0.082	0.957 (0.508-1.802)	0.892
AGR				
<1.24	1.000		1.000	
≥1.24	0.672 (0.447-1.010)	0.056	1.049 (0.623-1.765)	0.859
PNI				
<50.03	1.000		1.000	
≥50.03	0.339 (0.198-0.582)	**<0.001**	0.465 (0.236-0.916)	**0.027**

**Table 3:** Univariate and multivariate analyses of biomarkers for progression-free survival.

Characteristics	Univariate analysis	*p* value	Multivariate analysis	*p* value
	HR (95% CI)		HR (95% CI)	
Age (years)				
<60	1.000		1.000	
≥60	0.814 (0.545-1.216)	0.316	0.841 (0.533-1.327)	0.457
Sex				
Male	1.000		1.000	
Female	0.321 (0.153-0.676)	**0.003**	0.557 (0.154-1.030)	0.056
ECOG				
0-1	1.000		1.000	
2	2.986 (1.930-4.618)	**<0.001**	2.830 (1.707-4.691)	**<0.001**
LDH				
<240	1.000		1.000	
≥240	1.943 (0.640-2.790)	0.768	1.538 (0.941-1.905)	0.058
LYM				
<1.28	1.000		1.000	
≥1.28	0.599 (0.405-0.888)	**0.011**	0.752 (0.449-1.257)	0.277
TMB				
Low	1.000		1.000	
Intermediate	0.980 (0.680-1.412)	0.912	1.060 (0.789-1.423)	0.540
High	0.988 (0.754-1.296)	0.932	0.129 (0.766-1.665)	0.669
NLR				
<3.18	1.000		1.000	
≥3.18	1.397 (0.944-2.069)	0.095	0.687 (0.391-1.207)	0.191
PLR				
<201	1.000		1.000	
≥201	1.468 (0.999-2.158)	0.051	1.239 (0.678-2.262)	0.486
AGR				
<1.24	1.000		1.000	
≥1.24	0.556 (0.374-0.825)	**0.004**	0.769 (0.475-1.246)	0.286
PNI				
<50.03	1.000		1.000	
≥50.03	0.392 (0.237-0.647)	**<0.001**	0.493 (0.259-0.936)	**0.031**

## Data Availability

No data were used to support this study.
